# Chinese herbal injections for heart failure: A protocol for systematic review and network meta-analyses

**DOI:** 10.1097/MD.0000000000009973

**Published:** 2018-02-23

**Authors:** Fengwen Yang, Jiahan Zou, Long Ge, Jinhui Tian, Myeong Soo Lee, Ji Hee Jun, Junhua Zhang

**Affiliations:** a Evidence-Based Medicine Center, Tianjin University of Traditional Chinese Medicine, Tianjin; b Evidence-Based Medicine Center, Lanzhou University, Lanzhou, China; c Clinical Research Division, Korea Institute of Oriental Medicine, Daejeon, Republic of Korea.

**Keywords:** Chinese herbal injection, heart failure, network meta-analysis

## Abstract

**Background:**

Chinese herbal injections (CHIs) are commonly used for the treatment of heart failure in China. Due to the variety of CHIs used in clinic, selecting a suitable CHI for patients with heart failure is vital. This study aims to assess and compare the effect of different CHIs for heart failure using network meta-analysis (NMA).

**Methods:**

Six electronic databases, including PubMed, the Cochrane Central Register of Controlled Trials, Embase, China National Knowledge Infrastructure, Wanfang, and Chinese Biomedical Literature Database will be search from inception to January 2018. Randomized controlled trial (RCT) comparing CHI with another CHI will be included. The primary outcome will be changes in heart function classification and left ventricular ejection fraction. Risk of bias assessment of the included RCTs will be conducted according to the Cochrane Handbook 5.1.0. A Bayesian NMA will be performed using WinBUGS 14 software and the result figures will be generated using Stata 13 software. GRADE will be used to explore the quality of evidence.

**Results:**

The results of this NMA will be published in a peer-reviewed journal.

**Conclusion:**

Our study will generate evidence of CHIs for patients with heart failure and provide suggestions for Chinese medicine clinical practice or guideline.

**Ethics and dissemination:**

Ethics approval and patient consent are not required because this study is an NMA based solely on the published literature. The results of this NMA will be submitted to a peer-reviewed journal.

**Protocol registration number:**

PROSPERO CRD 42018086740

## 1 Introduction

Heart failure is a chronic disease exhibiting an unpredictable trajectory and an escalating symptom profile along with time.^[1]^ Almost 26 million people suffered from heart failure around the world.^[2]^ It is one of the most primary causes of hospitalization in people over 65 years,^[3]^ about 9.6% of patients with heart failure died and 19.4% rehospitalized for cardiovascular causes within 90 days of admission.^[4]^

According to the World Bank Development Indicators, the cost of heart failure in China during 2012 was up to $5.42 billion, ranking as the greatest healthcare burden among middle and low-income nations, and taking up 5.01% of total healthcare costs.^[5]^ Although Chinese herbal injections (CHIs) are not the mainstream for the treatment of heart failure in the world, they are widely used as a complementary and alternative therapy for patients with heart failure in China due to its rapid action, high bioavailability, and no digestive tract absorption.^[6]^

It is difficult to determine the superiority of CHIs using pairwise meta-analysis, network meta-analysis (NMA) has become increasingly popular in assessing different therapies through direct or indirect comparisons.^[7]^ It allows a comprehensive analysis of all studies that compared different kinds of interventions and rank them one by one.^[8,9]^ This study aims to evaluate the effect of different AHIs in the treatment of heart failure and to provide evidence for clinicians in the selection of AHIs.

## 2 Methods

### 2.1 Study registration

The study protocol has been registered on PROSPERO CRD 42018086740.

### 2.2 Eligibility criteria

#### 2.2.1 Type of study

Randomized controlled trials (RCTs) that investigated the effect of CHI for the treatment of heart failure will be included. No language limitation exists.

#### 2.2.2 Participants

Patients with heart failure, which should be confirmed according to the diagnostic standard.^[10–13]^ There are no limitations in age, gender, race, or nationality.

#### 2.2.3 Interventions

Interventions of CHI compare with another CHI are eligible, conventional pharmacotherapy (eg, cardiotonic, diuretic, vasodilators, ACEI, β-blocker, and so on) could be used in both groups or not. As the following 2 forms: CHI a versus CHI b; CHI a + conventional pharmacotherapy versus CHI b + conventional pharmacotherapy. Conventional pharmacotherapy in the 2 groups should be same.

#### 2.2.4 Outcomes

The primary outcomes include changes of heart function classification according to NYHA standard and left ventricular ejection fraction, the secondary outcome was adverse event. RCTs reporting on at least one primary outcome will be included.

### 2.3 Data source

Six electronic databases, including PubMed, the Cochrane Central Register of Controlled Trials, EMBASE, Chinese Biomedical Literature Database, China National Knowledge Infrastructure, and Wanfang Database will be searched from inceptions to January 2018. There was no language restriction in this study. Search strategy of PubMed was as follows:

#1 ((((((((((((((“Heart Failure”[MeSH Terms]) OR “Heart Failure”[Title/Abstract]) OR “Cardiac Failure”[Title/Abstract]) OR “Heart Decompensation”[Title/Abstract]) OR “Decompensation, Heart”[Title/Abstract]) OR “Right-Sided Heart Failure”[Title/Abstract]) OR “Right Sided Heart Failure”[Title/Abstract]) OR “Myocardial Failure”[Title/Abstract]) OR “Congestive Heart Failure”[Title/Abstract]) OR “Left-Sided Heart Failure”[Title/Abstract]) OR “Left Sided Heart Failure”[Title/Abstract])

#2 (((((Injections[MeSH Terms]) OR Injection[Title/Abstract]) OR Injectables[Title/Abstract]) OR Injectable[Title/Abstract]) OR Injections[Title/Abstract])))

#3 (((((“Medicine, Chinese Traditional”[MeSH Terms]) OR “Traditional Chinese Medicine”[Title/Abstract]) OR “Chinese Traditional Medicine”[Title/Abstract]) OR zhongyi[Title/Abstract]) OR zhongyao[Title/Abstract])

#4 #2 AND #3

#5 #1 AND #4

### 2.4 Study selection

Records from database searches will be managed by EndNote X7 software. The titles and abstracts of each record retrieved will be checked by 2 independent authors (FY and JZ) according to eligibility criteria. The full texts of potentially relevant studies will be retrieved for further assessment. Disagreements will be resolved by discussion or consultation of a 3rd author (JZ). A data spreadsheet will be created using Microsoft Excel 2007 to collect relevant information and data. The information, including author, year of publication, sample size, intervention, and outcome will be extracted from each study and entered into the spreadsheet.

### 2.5 Risk of bias (ROB) assessment

ROB assessment will be conducted by 2 independent authors (LG and JT). Disagreement will be resolved by discussion or consultation of Junhua Zhang. The ROB Tool in Cochrane Handbook 5.1.0 will be used to assess the potential ROB of included RCTs.^[14]^ Seven items are included in ROB: random sequence generation, allocation concealment, participant and personnel blinding, outcome assessment blinding, incomplete data, selective reporting, and other biases. Every item in ROB will be graded as low risk, high risk, or unclear risk.

### 2.6 Statistical analysis

#### 2.6.1 Pairwise meta-analyses

Pairwise meta-analyses will be carried out using random-effects model by Stata 13 software. Odds radio with 95% confidence interval (95% CI) will be presented for dichotomous data (heart function classification and adverse event) and continuous data (left ventricular ejection fraction) will be reported as mean difference with 95% CI. The χ^2^ test and *I*^2^ test will be conducted to assess the potential heterogeneity across the included studies. Sensitivity analyses, which was mainly according to the methodological qualities and sample size, will be carried out to investigate the source for potential heterogeneity if *I*^2^ >50% and *P* < 0.1. Subgroup analysis will also be performed if the heterogeneity was detected.

Begg and Egger funnel plot method through Stata 13 will be used to examine the publication bias when applicable.^[15,16]^

#### 2.6.2 Network meta-analyses

A Bayesian NMA will be performed by WinBUGS 14 (MRC Biostatistics Unit, Cambridge University, UK).^[17]^ We will use node splitting method to examine the inconsistency between direct and indirect comparisons if a loop connecting 3 or more arms exist.^[18]^ Surface under the cumulative ranking will be used to rank the CHIs,^[19]^ a larger surface under the cumulative ranking means a more effective intervention. Comparison-adjusted funnel plots will be conducted to assess the effects of the sample size on the results. All the result figures will be generated using Stata 13 software.

### 2.7 Quality of evidence

The quality of evidence for the main outcomes will be assessed using the Grading of Recommendations Assessment, Development and Evaluation,^[20]^ which contains 4 levels: high level, moderate level, low level, and very low level. Flaws in study design, inconsistency, imprecision, indirectness, and publication bias will be investigated.

## 3 Discussion

This NMA will summarize the direct and indirect evidence through providing a ranking of the CHIs for heart failure. To the best of our knowledge, this is the first NMA to comprehensively evaluate and compare the effect of different CHIs for heart failure, although a few studies have assessed the effect of the individual CHI.^[6,21,22]^ We hope the results of this NMA will provide the clinical recommendation for patients with heart failure in Chinese medicine clinical practice or guideline.

This protocol is designed in adherence to guideline for NMA protocols^[23]^ and will be conducted and reported strictly according to the PRISMA extension statement for NMAs.^[24]^

## References

[1] Roger VL Go AS Lloyd-Jones DM Heart disease and stroke statistics-2012 update. Circulation 2012;125:e2–20. 22179539 10.1161/CIR.0b013e31823ac046 PMC4440543

[2] Pocock SJ Wang D Pfeffer MA Predictors of mortality and morbidity in patients with chronic heart failure. Eur Heart J 2006;27:65–75. 16219658 10.1093/eurheartj/ehi555

[3] Go AS Mozaffarian D Roger VL American Heart Association Statistics Committee and Stroke Statistics Subcommittee. Heart disease and stroke statistics-2013 update: a report from the American Heart Association. Circulation 2013;127:e6–245. 23239837 10.1161/CIR.0b013e31828124ad PMC5408511

[4] Gheorghiade M Pang PS Ambrosy AP A comprehensive, longitudinal description of the in-hospital and post-discharge clinical, laboratory, and neurohormonal course of patients with heart failure who die or are re-hospitalized within 90 days: analysis from the EVEREST trial. Heart Fail Rev 2012;17:485–509. 21932146 10.1007/s10741-011-9280-0

[5] Cook C Cole G Asaria P The annual global economic burden of heart failure. Int J Cardiol 2014;171:368–76. 24398230 10.1016/j.ijcard.2013.12.028

[6] Wang K Wu J Duan X Huangqi injection in the treatment of chronic heart failure: a systematic review and meta-analysis. Medicine 2017;96:e8167. 28953668 10.1097/MD.0000000000008167 PMC5626311

[7] Bafeta A Trinquart L Seror R Reporting of results from network meta-analyses: methodological systematic review. BMJ 2014;348:g1741. 24618053 10.1136/bmj.g1741 PMC3949412

[8] Higgins JP Whitehead A Borrowing strength from external trials in a meta-analysis. Stat Med 1996;15:2733–49. 8981683 10.1002/(SICI)1097-0258(19961230)15:24<2733::AID-SIM562>3.0.CO;2-0

[9] White IR Network meta-analysis. Stata J 2015;15:951–85.

[10] McMurray JJ Adamopoulos S Anker SD ESC Guidelines for the diagnosis and treatment of acute and chronic heart failure 2012: The Task Force for the Diagnosis and Treatment of Acute and Chronic Heart Failure 2012 of the European Society of Cardiology. Developed in collaboration with the Heart Failure Association (HFA) of the ESC. Eur Heart J 2012;33:1787–847. 22611136 10.1093/eurheartj/ehs104

[11] Yancy CW Jessup M Bozkurt B 2013 ACCF/AHA guideline for the management of heart failure: a report of the American College of Cardiology Foundation/American Heart Association Task Force on practice guideline. Circulation 2013;128:e240–327. 23741058 10.1161/CIR.0b013e31829e8776

[12] Chinese Society of Cardiology, Editorial Committee of Chinese Journal of Cardiology. Guidelines for the diagnosis and treatment of heart failure in China. Chin J Cardiol 2014;42:98–122.

[13] Zheng YY Clinical Guideline of New Drugs for Traditional Chinese Medicine. Beijing: Chin Med Sci and Tech Press; 2002.

[14] Higgins JPT Altman DG Sterne JAC Chapter 8: Assessing risk of bias in included studies. In: Higgins, J.P.T., Green, S., editors. Cochrane Handbook for Systematic Reviews of Interventions version 5.1.0 (updated March 2011): The Cochrane collaboration. Available from www.chochrane-handbook.org. 2011, pp. 243–296.

[15] Begg CB Mazumdar M Operating characteristics of a rank correlation test for publication bias. Biometrics 1994;50:1088. 7786990

[16] Egger M Davey Smith G Schneider M Bias in meta-analysis detected by a simple, graphical test. BMJ 1997;315:629–34. 9310563 10.1136/bmj.315.7109.629 PMC2127453

[17] Lunn DJ Thomas A Best N WinBUGS – a Bayesian modeling framework: concepts, structure, and extensibility. Stat Comput 2000;10:325–37.

[18] Lu G Ades AE Combination of direct and indirect evidence in mixed treatment comparisons. Stat Med 2004;23:3105–24. 15449338 10.1002/sim.1875

[19] Salanti G Ades AE Ioannidis JP Graphical methods and numerical summaries for presenting results from multiple-treatment meta-analysis: an overview and tutorial. J Clin Epidemiol 2011;64:163–71. 20688472 10.1016/j.jclinepi.2010.03.016

[20] Puhan MA Schünemann HJ Murad MH A GRADE Working Group approach for rating the quality of treatment effect estimates from network meta-analysis. BMJ 2014;349:g5630. 25252733 10.1136/bmj.g5630

[21] Xian S Yang Z Lee J A randomized, double-blind, multicenter, placebo-controlled clinical study on the efficacy and safety of Shenmai injection in patients with chronic heart failure. J Ethnopharmacol 2016;186:136–42. 27045864 10.1016/j.jep.2016.03.066

[22] Shi LW Xie YM Liao X Shenmai injection as an adjuvant treatment for chronic cor pulmonale heart failure: a systematic review and meta-analysis of randomized controlled trials. BMC Complement Altern Med 2015;15:418. 26603978 10.1186/s12906-015-0939-2 PMC4659214

[23] Shamseer L Moher D Clarke M Preferred reporting items for systematic review and meta-analysis protocols (PRISMA-P) 2015: elaboration and explanation. BMJ 2015;349:g7647. 10.1136/bmj.g7647 25555855

[24] Hutton B Salanti G Caldwell DM The PRISMA extension statement for reporting of systematic reviews incorporating network meta-analyses of health care interventions: checklist and explanations. Ann Intern Med 2015;162:777–84. 26030634 10.7326/M14-2385

